# Supplementary material for: Gamma-Delta T-Cell Phenotype and Function in DAA-Treated HIV-HCV Co-Infected and HCV-Mono-Infected Subjects
Source: Viruses. 2022 Jul 22;14(8):1594. doi: 10.3390/v14081594 (PMC9329743; doi:10.3390/v14081594)
Supplement: Supplementary file 1 [file viruses-14-01594-s001.zip › viruses-1748780-supplementary.pdf]

Figure S1: Gating stratgey for Th17 and Treg cells

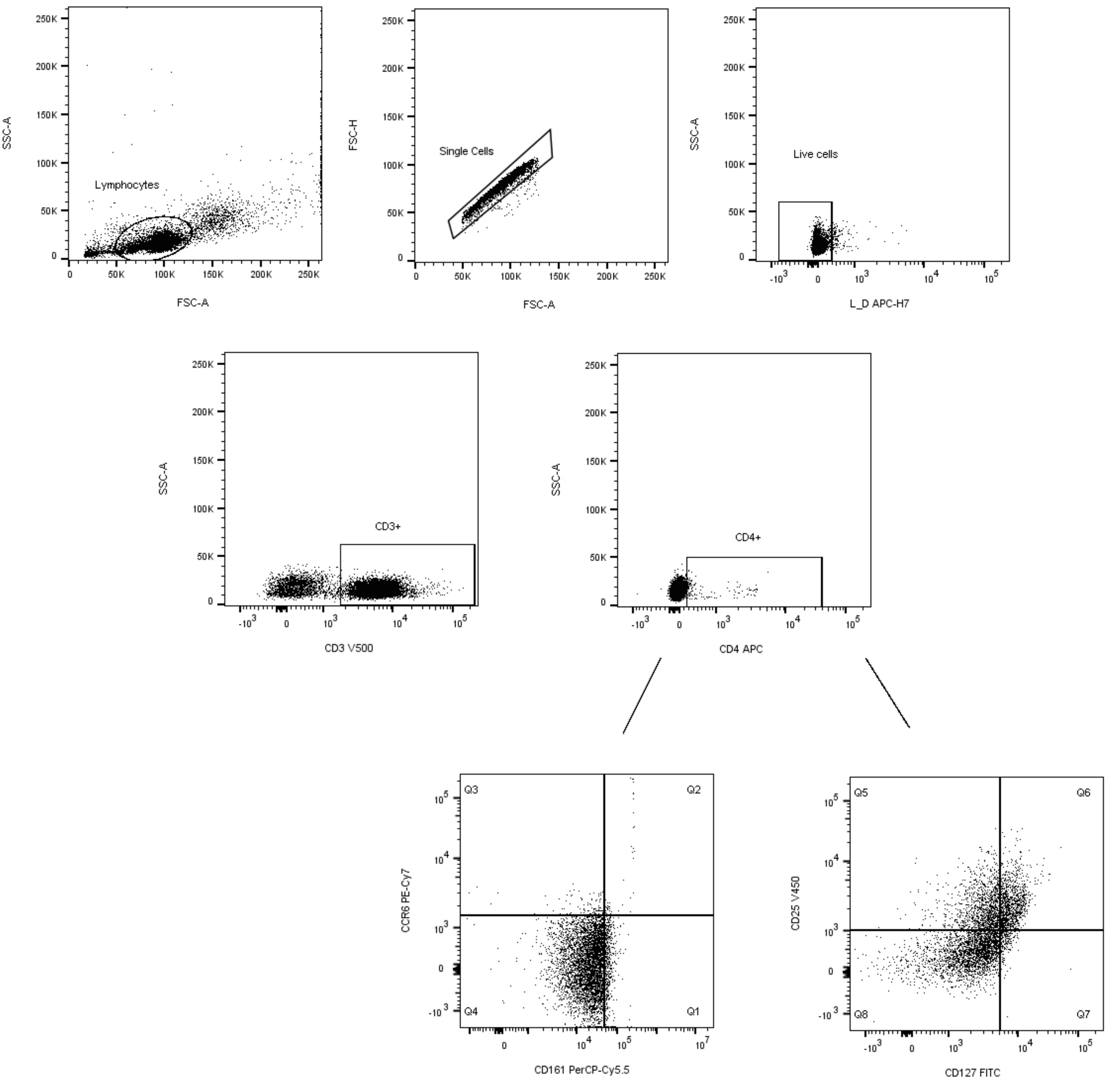

Representative plots illustrate the gating strategy. Lymphocytes, single cells, live cells, CD3 and CD4 T-cells were identified. Th17 (CCR6+ CD161+) and Treg (CD25+ CD127-) cells were identified in the CD4+ T cells.

Figure S2: Gating stratgey for γδ cells

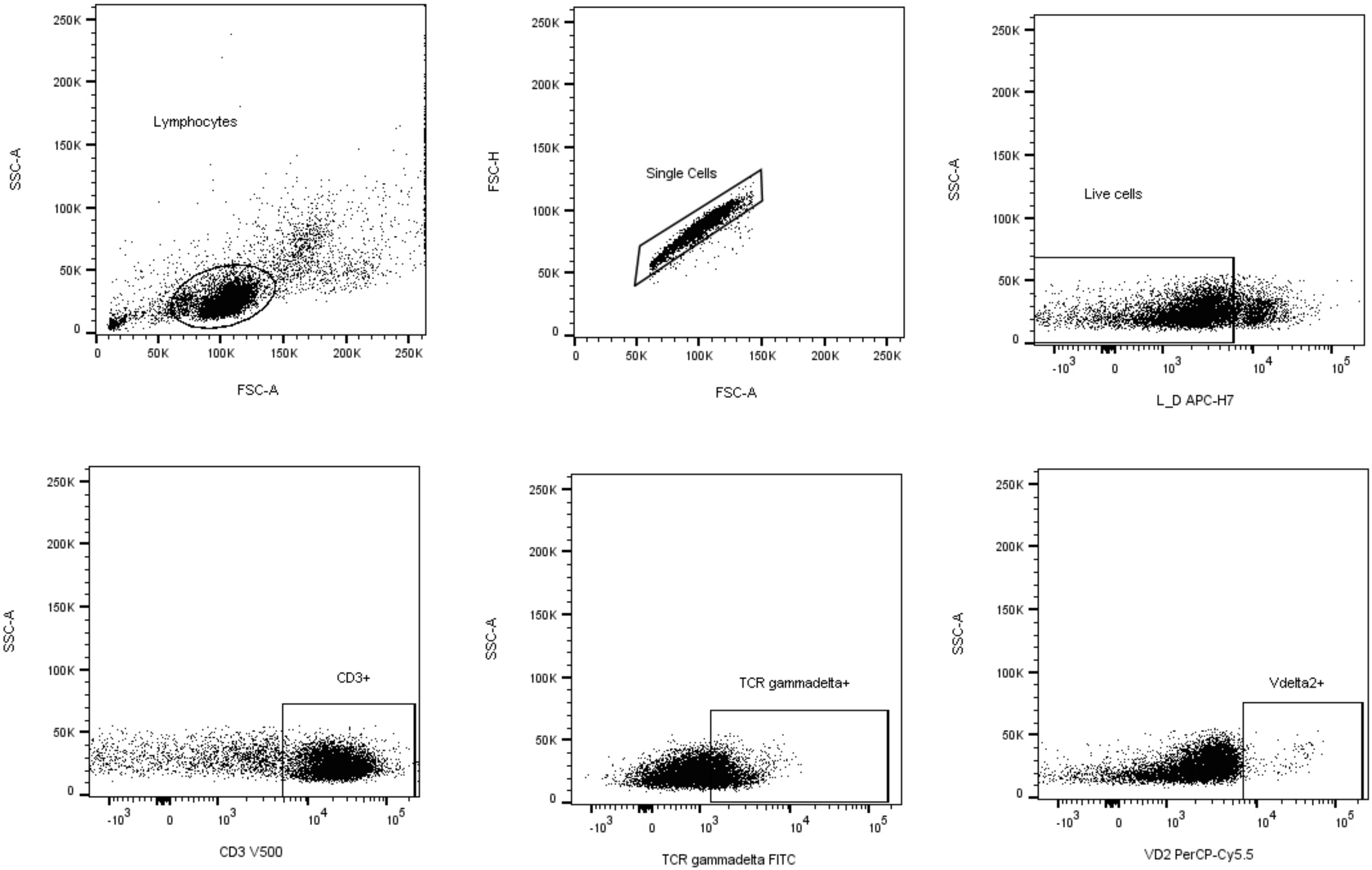

Representative plots illustrate the gating strategy. Lymphocytes, single cells, live cells, CD3, TCRγδ and Vδ2 cells were identified

Figure S3: Gating stratgey for B-cells

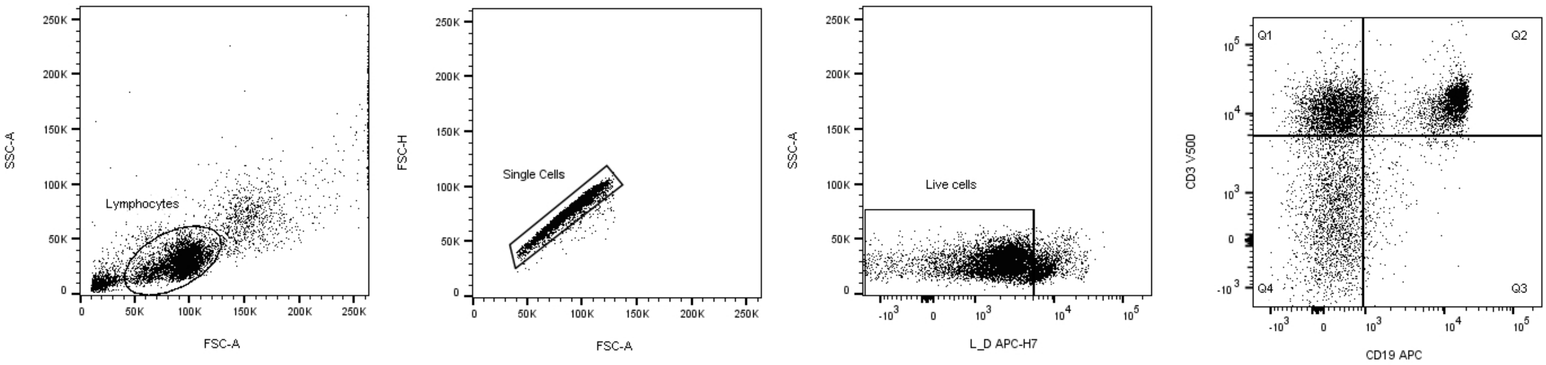

Representative plots illustrate the gating strategy. Lymphocytes, single cells, live cells and B-cells (CD3- CD19+) were identified.
